# The Host-Specific Intestinal Microbiota Composition Impacts *Campylobacter coli* Infection in a Clinical Mouse Model of Campylobacteriosis

**DOI:** 10.3390/pathogens9100804

**Published:** 2020-09-29

**Authors:** Markus M. Heimesaat, Claudia Genger, Sigri Kløve, Dennis Weschka, Soraya Mousavi, Stefan Bereswill

**Affiliations:** Institute of Microbiology, Infectious Diseases and Immunology, Charité-University Medicine Berlin, Corporate Member of Freie Universität Berlin, Humboldt-Universität zu Berlin and Berlin Institute of Health, 12203 Berlin, Germany; claudia.genger@charite.de (C.G.); sigri.klove@charite.de (S.K.); dennis.weschka@charite.de (D.W.); soraya.mousavi@charite.de (S.M.); stefan.bereswill@charite.de (S.B.)

**Keywords:** *Campylobacter coli*, campylobacteriosis model, host–pathogen interaction, microbiota-depleted IL-10^−/−^ mice, intestinal immunopathology, bacterial colonization capacity, host-specific intestinal microbiota, fecal microbiota transplantation, colonization resistance

## Abstract

Human *Campylobacter*-infections are progressively rising globally. However, the molecular mechanisms underlying *C. coli*–host interactions are incompletely understood. In this study, we surveyed the impact of the host-specific intestinal microbiota composition during peroral *C. coli* infection applying an established murine campylobacteriosis model. Therefore, microbiota-depleted IL-10^−/−^ mice were subjected to peroral fecal microbiota transplantation from murine versus human donors and infected with *C. coli* one week later by gavage. Irrespective of the microbiota, *C. coli* stably colonized the murine gastrointestinal tract until day 21 post-infection. Throughout the survey, *C. coli*-infected mice with a human intestinal microbiota displayed more frequently fecal blood as their murine counterparts. Intestinal inflammatory sequelae of *C. coli*-infection could exclusively be observed in mice with a human intestinal microbiota, as indicated by increased colonic numbers of apoptotic epithelial cells and innate as well as adaptive immune cell subsets, which were accompanied by more pronounced pro-inflammatory cytokine secretion in the colon and mesenteric lymph nodes versus mock controls. However, in extra-intestinal, including systemic compartments, pro-inflammatory responses upon pathogen challenge could be assessed in mice with either microbiota. In conclusion, the host-specific intestinal microbiota composition has a profound effect on intestinal and systemic pro-inflammatory immune responses during *C. coli* infection.

## 1. Introduction

*Campylobacter* infections represent frequent causes of human enteritis with rising prevalence worldwide. The majority of campylobacteriosis cases are attributed to *Campylobacter jejuni* and *Campylobacter coli* [1,2], and pathogen-induced clinical symptoms are characterized by watery or bloody, inflammatory diarrhea, abdominal pain, and fever [3,4]. While *Campylobacter* infections are usually self-limiting and mostly require symptomatic treatment, antimicrobial interventions are only recommended in severe cases, particularly in immuno-compromised individuals [4,5]. In rare cases, primary campylobacteriosis triggers long-term post-infectious sequelae, such as Guillain-Barré syndrome, reactive arthritis, and chronic inflammatory intestinal illnesses, including coeliac disease, irritable bowel syndrome, and chronic inflammatory bowel diseases [4,5,6].

*Campylobacter* are present in both urban and agricultural environments, and have been isolated from water sources and from intestinal contents of vertebrates including wild birds and mammals [7,8,9]. The main mode of human *Campylobacter* infections is ingestion of contaminated water, milk and undercooked meat derived from livestock, mostly chickens and other poultry. Given that the bacteria are part of the commensal gastrointestinal microbiota [5,10], *C. jejuni* and *C. coli* share the same reservoirs, while the prevalence amongst species varies. *C. coli*, for instance, have been found in swine and sheep at high frequencies [9,11,12].

Given that *C. coli* infections far less frequently cause human enteritis than *C. jejuni* [13], *Campylobacter* research has been focused mostly on the latter. Therefore, our knowledge regarding the molecular mechanisms of *C. coli*–host interactions is very limited. However, the burdens that *C. coli* infections cause to the public health and the socioeconomic systems worldwide should not be underscored. In fact, depending on the geographical location, *C. coli* are responsible for up to 25% of reported campylobacteriosis cases [14].

Detailed analyses of the molecular mechanisms underlying the immunopathogenesis of campylobacteriosis have been hampered by the lack of appropriate experimental infection and inflammation models. Particularly, conventional laboratory mice can hardly be infected by *C. jejuni* due to the colonization resistance that is caused by the mouse-specific intestinal microbiota composition, preventing the murine host from invading pathogens [15,16]. Therefore, researchers successfully abrogated the colonization resistance by antibiotic treatment of mice [16,17,18]. Upon peroral *C. jejuni* infection, microbiota depleted wildtype mice were effectively colonized by the pathogens but did not develop overt clinical signs of intestinal inflammation due to their resistance to pathogenic lipooligosaccharide (LOS), constituting a major pathogenicity factor of *Campylobacter* [19]. The recent progress that has been made in overcoming these fundamental obstacles in *Campylobacter* research is based upon the modification of the host-specific intestinal microbiota composition and sensitization of the murine host to *C. jejuni*-LOS by genetic or dietary modulations [19]. We and others have applied microbiota-depleted interleukin-10-deficient (IL-10^−/−^) mice, for instance, that develop *C. jejuni*-induced intestinal inflammation resembling clinical and immunopathological key features of human campylobacteriosis [16,20,21,22].

Our recent research provided further evidence that the host-specific intestinal microbiota composition plays a pivotal role in the resistance of mice against *C. jejuni* colonization since *C. jejuni* was able to establish in the gastrointestinal tract of mice with a human intestinal microbiota, whereas mice with a murine intestinal microbiota were completely protected from pathogenic infection [16,23].

In order to extend these findings to *C. coli*, we have previously surveyed the gastrointestinal colonization properties of *C. coli* in conventional wildtype mice. Following peroral infection with a *C. coli* strain that had been isolated from a human patient presenting with bloody diarrhea, the bacteria were able to colonize the gastrointestinal tract of mice with a conventional intestinal microbiota until day 21 post-challenge, which was, however, not the case when perorally applying the closely related *C. jejuni* bacteria [24]. This complete absence of the physiological colonization resistance against *C. coli* in conventional mice prompted us in our actual study to further unravel the triangle relationship (“ménage-à-trois”) between the pathogen, the vertebrate host immunity, and the intestinal microbiota of murine versus human origin in a clinical acute campylobacteriosis model by using microbiota-depleted IL-10^−/−^ mice.

## 2. Results

### 2.1. Association of Microbiota-Depleted IL-10^−/−^ Mice with a Murine or Human Fecal Microbiota Prior C. coli Infection

**Figure 1:**
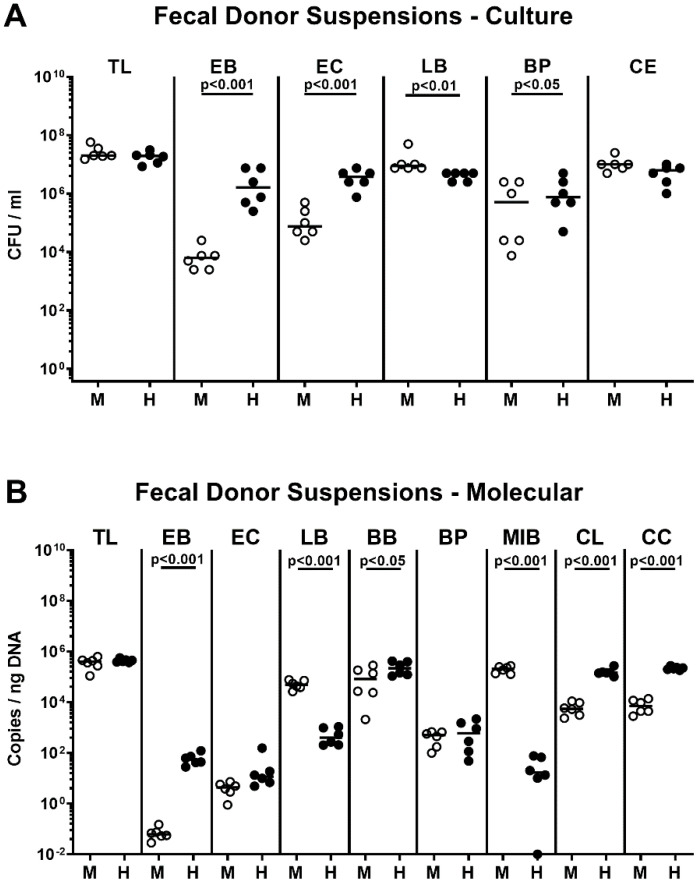
Bacterial composition of human and murine donor suspensions used for fecal microbiota transplantation. Immediately before fecal microbiota transplantations on three consecutive days (i.e., days -7, -6, -5), the bacterial compositions of respective donor suspensions derived from murine (M; open circles) or human (H; closed circles) donors were quantitatively assessed by (**A**) culture and (**B**) culture-independent 16S rRNA-based methods and expressed as colony forming units per ml (CFU/mL) and copies/ng DNA, respectively. Medians (black bars) and significance levels (*p*-values) assessed by the Mann–Whitney U test are indicated. Data were pooled from two independent experiments. TL: total bacterial load; EB: enterobacteria; EC: enterococci; LB: lactobacilli; BB: bifidobacteria; BP: *Bacteroides/Prevotella* species; MIB: *Mouse Intestinal Bacteroides*; CE: *Clostridium/Eubacterium* species; CL: *Clostridium leptum* group; CC: *Clostridium coccoides* group (n = 6).

In order to assess the impact of the host-specific intestinal microbiota on *C. coli* infection and subsequent inflammatory responses, microbiota-depleted IL-10^−/−^ mice were perorally subjected to fecal microbiota transplantations (FMT) from human or murine donors on three occasions (namely, days -7, -6, and -5; Supplementary Figure S1). Cultural as well as culture-independent (i.e., 16S rRNA-based) analyses of the main intestinal bacterial groups abundant in the fecal donor suspensions revealed distinct host-specific differences in microbiota composition as indicated by higher loads of enterobacteria, enterococci, bifidobacteria, *Bacteroides/Prevotella* species (spp.)., *Clostridium leptum*, and *Clostridium coccoides* groups, but lower lactobacilli and *Mouse Intestinal Bacteroides* counts in human versus murine donor suspensions (*p* < 0.05–0.001; Figure 1).

**Figure 2:**
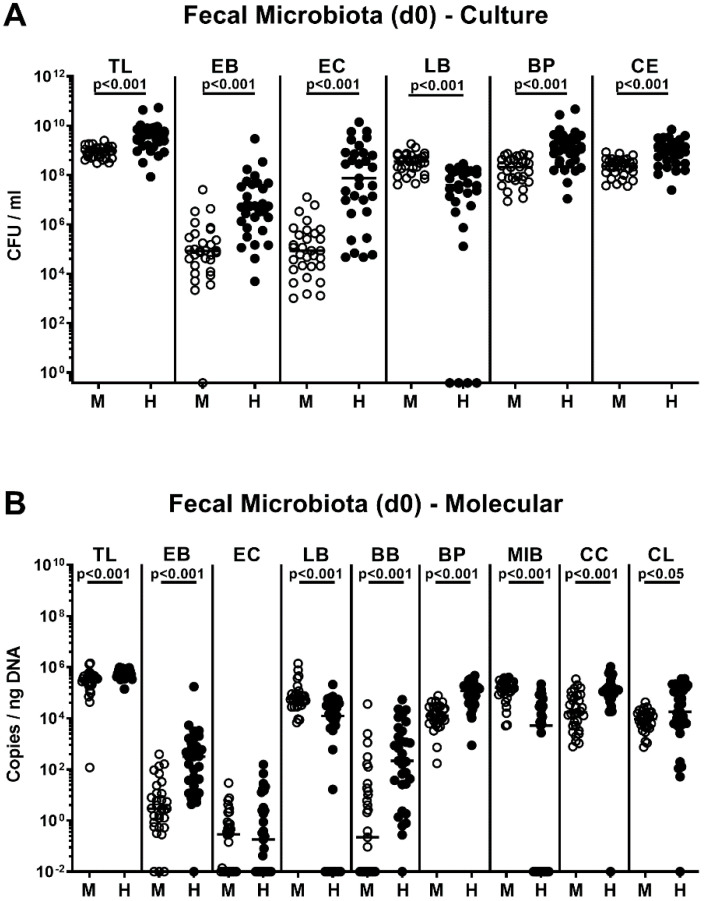
Intestinal microbiota composition of microbiota-depleted IL-10^−/−^ mice that had been challenged with human or murine microbiota transplantations immediately before *C. coli* infection. Immediately before *C. coli* infection (i.e., day (d) 0), and seven days after the first of three consecutive murine (M; open circles) versus human (H; closed circles) fecal microbiota transplantations, the fecal microbiota composition was quantitatively assessed by (**A**) culture and (**B**) culture-independent 16S rRNA-based (molecular) methods and expressed as colony forming units per ml (CFU/mL) and copies/ng DNA, respectively. Medians (black bars) and significance levels (*p*-values) assessed by the Mann–Whitney U test are indicated. Data were pooled from four independent experiments. TL: total bacterial load; EB: enterobacteria; EC: enterococci; LB: lactobacilli; BB: bifidobacteria; BP: *Bacteroides/Prevotella* species; MIB: *Mouse Intestinal Bacteroides*; CE: *Clostridium/Eubacterium* species; CL: *Clostridium leptum* group; CC: *Clostridium coccoides* group (n = 31).

Immediately before the first of two peroral *C. coli* infections on days 0 and 1, the bacterial colonization efficacies following FMT that had been started 7 days before was quantitatively assessed again by culture and culture-independent analyses. Like in case of the bacterial donor suspensions, pronounced host-specific differences in fecal microbiota compositions could be observed as indicated by higher total eubacterial loads and by higher numbers of enterobacteria, enterococci, bifidobacteria, *Bacteroides/Prevotella* spp., *Clostridium leptum*, and *Clostridium coccoides* groups in human as compared to mouse microbiota-associated mice (*p* < 0.05–0.001), whereas lactobacilli and *Mouse Intestinal Bacteroides* loads were lower in the former versus the latter (*p* < 0.001; Figure 2). Hence, distinct host-specific differences in the intestinal microbiota composition could be assessed immediately before *C. coli* infection of secondary abiotic IL-10^−/−^ mice that had been challenged with human or murine FMT.

### 2.2. Fecal Pathogen Shedding after Oral C. coli Infection of Microbiota-Depleted IL-10^−/−^ Mice that Had Been Subjected to Human or Murine Fecal Microbiota Transplantations

**Figure 3:**
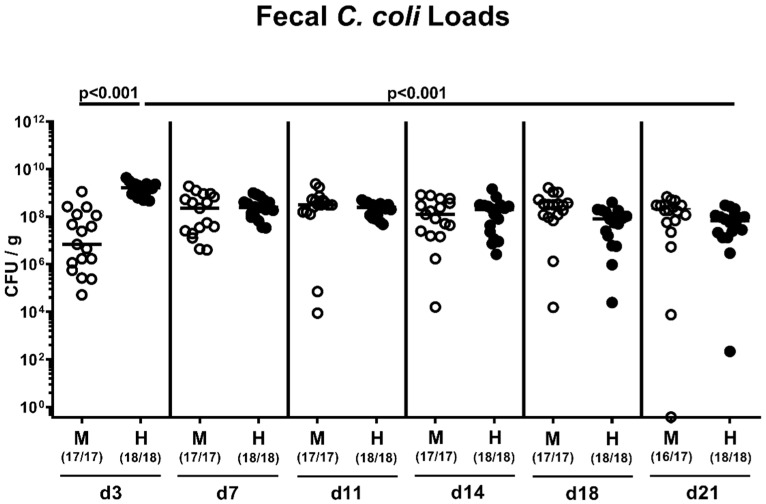
Fecal *C. coli* shedding over time upon oral infection of microbiota-depleted IL-10^−/−^ mice that had been challenged with human or murine fecal microbiota transplantations. On days -7, -6, and -5, mice were subjected to murine (M; open circles) or human (H; closed circles) fecal microbiota transplantations. On days 0 and 1, microbiota-associated mice were then perorally infected with a *C. coli* patient isolate and fecal bacterial loads were quantitated in each mouse over time post-infection by culture (expressed as colony forming units per g; CFU/g). Medians (black bars), significance levels (*p*-values calculated by the Kruskal–Wallis test and Dunn’s post-correction and by the Mann–Whitney U test comparing *C. coli* loads in mice following murine versus human FMT at identical time points post-infection) and numbers of culture-positive animals out of the total number of mice under investigation (in parentheses) are indicated. Results from four experiments were pooled.

We then performed a kinetic survey of the fecal pathogen loads following peroral infection with 10^8^ viable *C. coli* that had been derived from a patient presenting with bloody diarrhea on two consecutive days (i.e., on days 0 and 1; Supplementary Figure S1) by gavage. Regardless of whether the mice had been subjected to human or murine FMT, *C. coli* could stably establish within the intestinal tract until the end of the survey (namely, 21 days post-infection (p.i.)) with median loads of approximately 10^8^ colony forming units (CFU) per g feces (Figure 3). As early as 48 h after the latest *C. coli* infection, pathogen loads were slightly higher in human versus murine intestinal microbiota-associated mice (*p* < 0.001; Figure 3). Furthermore, in mice that had been challenged with a human fecal microbiota, *C. coli* loads decreased by less than two log orders from day 3 till day 21 p.i. (*p* < 0.001; Figure 3).

**Figure 4:**
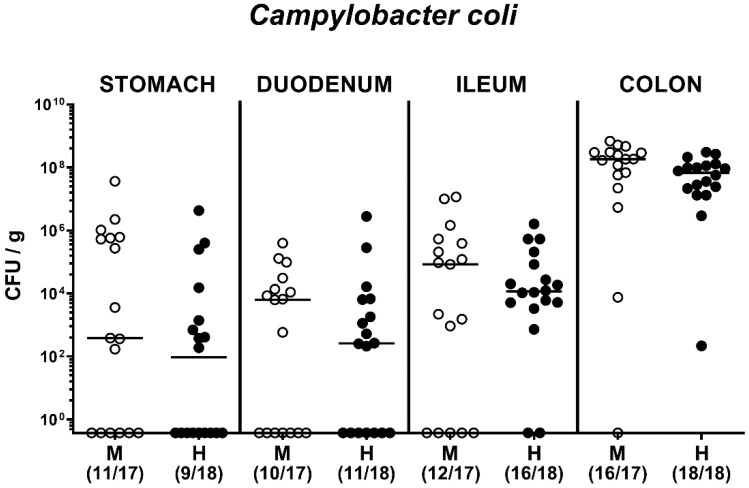
Gastrointestinal *C. coli* loads upon oral infection of microbiota-depleted IL-10^−/−^ mice that had been challenged with human or murine fecal microbiota transplantations. On days -7, -6, and -5, mice were subjected to murine (M; open symbols) or human (H; closed circles) fecal microbiota transplantations. On day 21 following peroral infection with a *C. coli* patient isolate on day 0 and day 1, the pathogen burdens were quantitated in gastrointestinal compartments applying culture and expressed as colony forming units per g (CFU/g). Numbers of culture-positive animals out of the total number of mice under investigation (in parentheses) and medians (black bars) are indicated. Results from four experiments were pooled.

Upon necropsy on day 21 p.i., we quantitated *C. coli* alongside the gastrointestinal tract (namely, the stomach, duodenum, ileum, and colon) and observed (from the intestinal microbiota composition independent) comparable loads in respective compartments with highest counts in the large intestines (Figure 4). Hence, *C. coli* was able to establish within the gastrointestinal tract of microbiota-depleted mice that had been challenged with human or murine FMT.

### 2.3. Kinetic Assessment of Clinical Conditions Following Peroral C. coli Infection of Microbiota-Depleted IL-10^−/−^ Mice That Had Been Subjected to Human or Murine Fecal Microbiota Transplantations

We next surveyed the clinical conditions over time post-pathogen challenge by a standardized clinical scoring system quantitatively assessing wasting, stool consistency, and the occurrence of fecal blood (Supplementary Figure S2). At days 7 and 21 following *C. coli* infection, mice harboring a human, but not murine intestinal microbiota displayed higher clinical scores as compared to time points prior to infection (i.e., days -7 and 0; *p* < 0.01; Supplementary Figure S2D), whereas at the end of the survey, 44.4% and 35.3% of infected mice with a human and murine microbiota respectively, displayed any *C. coli*-induced clinical signs (Supplementary Figure S1B,D). Mice from respective mock cohorts, however, were clinically uncompromised (Supplementary Figure S2A,C).

**Figure 5:**
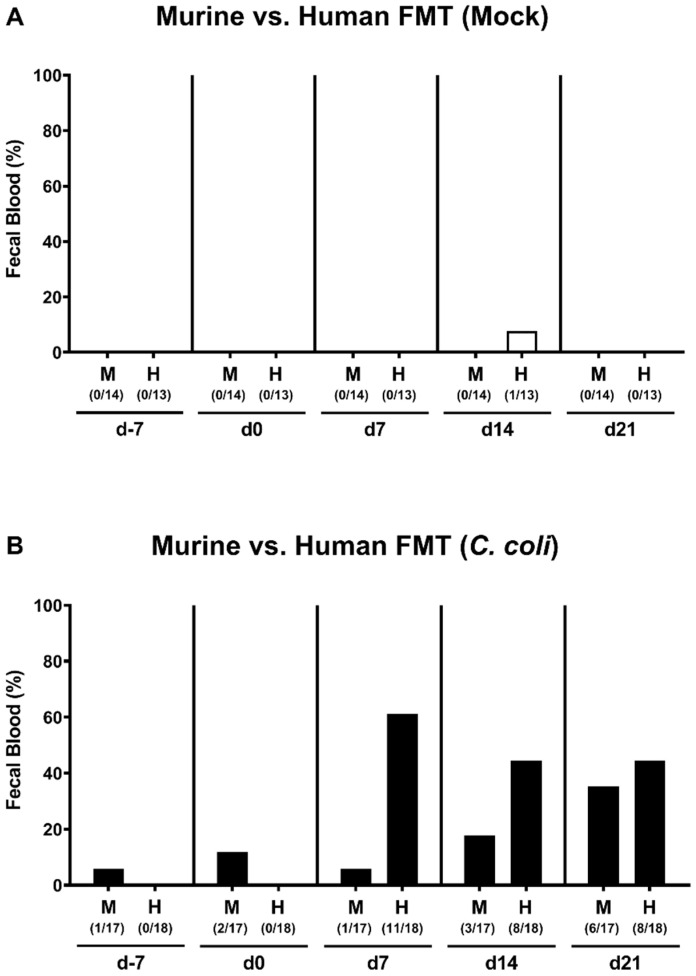
Occurrence of fecal blood over time following *C. coli* infection of microbiota-depleted IL-10^−/−^ mice that had been challenged with human or murine fecal microbiota transplantations. On days -7, -6, and -5, mice were subjected to murine or human fecal microbiota transplantation (FMT) and challenged with a *C. coli* patient isolate (closed bars; (**B**)) or vehicle as mock controls (open bars; (**A**)) on days 0 and 1 by gavage. Bars indicate the cumulative frequencies of fecal blood as assessed by the Guajak method (in %). Numbers of fecal blood-positive animals out of the total number of mice under investigation are given (in parentheses). Results from four experiments were pooled.

When assessing the occurrence of blood in fecal samples (Figure 5), 61.1% of mice harboring a human intestinal microbiota, but only 5.9% from the murine microbiota cohort, displayed fecal blood on day 7 p.i., whereas later on, on day 21 p.i., respective fecal blood positivity rates were 44.4% and 35.3% in the former as compared to the latter (Figure 5B). In none of the mock control mice any fecal blood was detected at days 7 and 21 p.i. (Figure 5A). Hence, particularly during the early phase of *C. coli* infection, mice harboring a human as opposed to a murine intestinal microbiota displayed fecal blood.

### 2.4. Intestinal Histopathological, Apoptotic, and Pro-Inflammatory Immune Responses upon Peroral C. coli Infection of Microbiota-Depleted IL-10^−/−^ Mice That Had Been Subjected to Human or Murine Fecal Microbiota Transplantations

**Figure 6:**
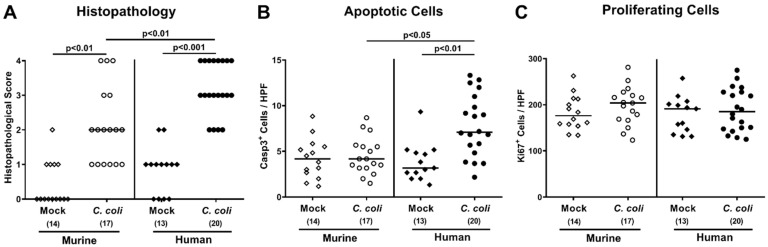
Colonic histopathological, epithelial apoptotic, and proliferative cell responses upon oral *C. coli* infection of microbiota-depleted IL-10^−/−^ mice that had been challenged with human or murine fecal microbiota transplantations. On days (d) -7, -6, and -5, mice were subjected to murine (open symbols) or human (closed symbols) fecal microbiota transplantations and challenged with a *C. coli* patient isolate (circles) or vehicle as mock controls (diamonds) on days 0 and 1 by gavage. On day 21 post-infection, (**A**) histopathological changes were quantitated in large intestinal paraffin sections according to a standardized histopathological scoring protocol. Furthermore, the average numbers of (**B**) apoptotic (Casp3+) and (**C**) proliferating (Ki67+) cells were determined in colonic epithelia out of 6 high-power fields (HPF, 400× magnification) per mouse. Medians (black bars), significance levels (*p*-values calculated by the Kruskal–Wallis test and Dunn’s post-correction), and total numbers of mice under investigation (in parentheses) are shown. Results from four experiments were pooled.

We next quantitated large intestinal histopathological changes using an established histopathological scoring system [25]. At day 21 following *C. coli* infection of human or murine intestinal microbiota-associated mice, higher histopathological scores could be determined as compared to respective mock control animals (*p* < 0.01 and *p* < 0.001, respectively) which were higher in the former versus the latter (*p* < 0.01; Figure 6A). Since apoptosis is considered another valuable marker for inflammatory responses in the intestinal tract [16], we quantitated cleaved caspase3^+^ cell numbers in colonic epithelia by in situ immunohistochemistry. At day 21 following *C. coli* infection, mice with a human, but not a murine intestinal microbiota, displayed elevated numbers of apoptotic colonic epithelial cells as compared to respective mock control counterparts (*p* < 0.01 versus mock; Figure 6B and Supplementary Figure S3B), whereas Ki67^+^ cells counts indicative for cell proliferation/regeneration were comparable in respective groups (not significant (n.s.); Figure 6C and Supplementary Figure S3C).

**Figure 7:**
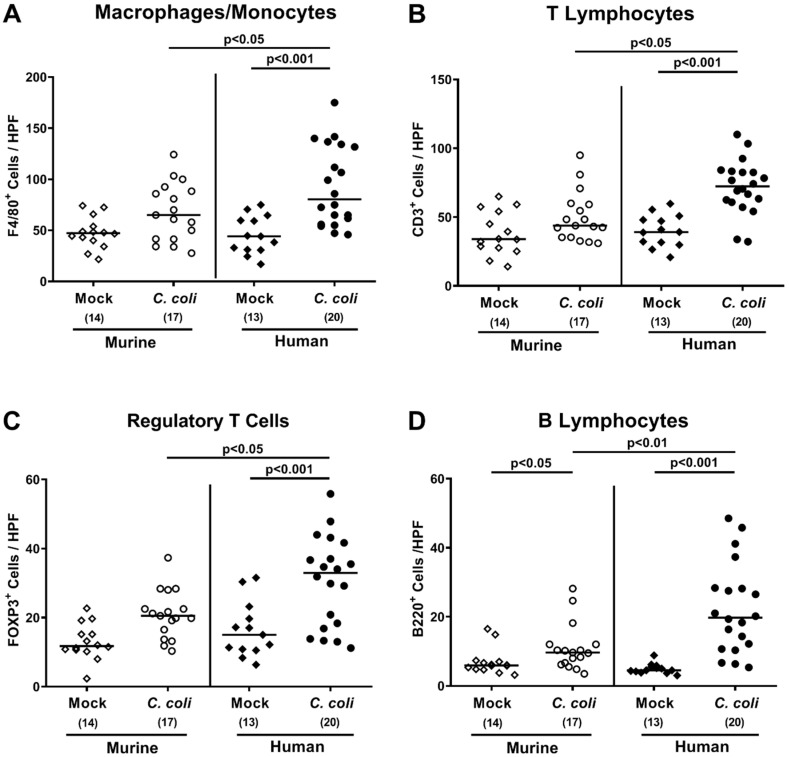
Colonic immune cell responses upon oral *C. coli* infection of microbiota-depleted IL-10^−/−^ mice that had been challenged with human or murine fecal microbiota transplantations. On days -7, -6, and -5, mice were subjected to murine (open symbols) or human (closed symbols) fecal microbiota transplantations and challenged with a *C. coli* patient isolate (circles) or vehicle as mock controls (diamonds) on days 0 and 1 by gavage. On day 21 post-infection, the average numbers of (**A**) macrophages and monocytes (F4/80+), (**B**) T lymphocytes (CD3+), (**C**) regulatory T cells (FOXP3+), and (**D**) B lymphocytes (B220+) were determined in the large intestinal mucosa and lamina propria out of 6 high-power fields (HPF, 400x magnification) per mouse. Medians (black bars), significance levels (*p*-values calculated by the Kruskal–Wallis test and Dunn’s post-correction), and total numbers of mice under investigation (in parentheses) are shown. Results from four experiments were pooled.

We further surveyed innate as well as adaptive immune cell responses in the large intestines following *C. coli* infection, again by quantitative in situ immunohistochemical analyses of colonic paraffin sections. Like the apoptotic epithelial cell counts, *C. coli* infection of mice with a human, but not with a murine intestinal microbiota, was paralleled by elevated numbers of F4/80^+^ macrophages and monocytes, of CD3^+^ T lymphocytes, of FOXP3^+^ regulatory T cells, and of B220^+^ B lymphocytes in the colonic mucosa and lamina propria versus mock controls at day 21 p.i. (*p* < 0.001; Figure 7 and Supplementary Figure S3D–G).

**Figure 8:**
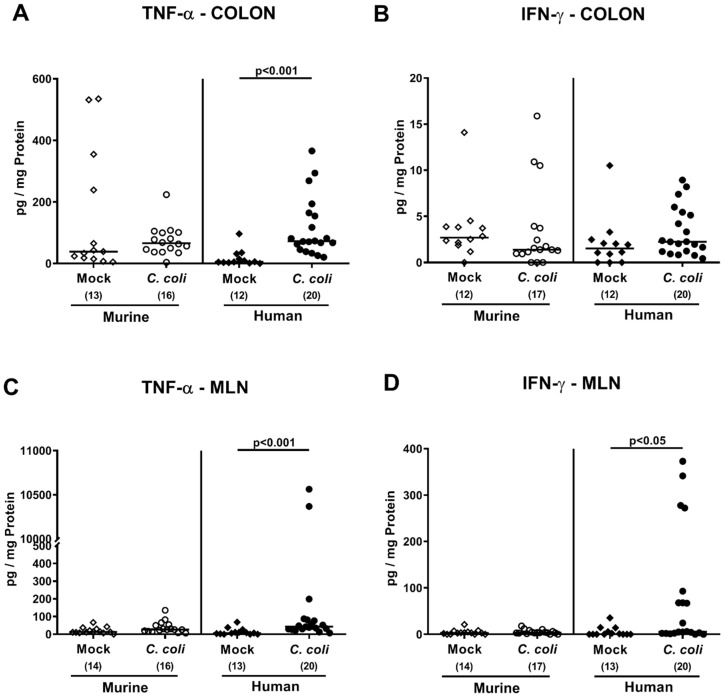
Intestinal pro-inflammatory cytokine secretion upon oral *C. coli* infection of microbiota-depleted IL-10^−/−^ mice that had been challenged with human or murine fecal microbiota transplantations. On days -7, -6, and -5, mice were subjected to murine (open symbols) or human (closed symbols) fecal microbiota transplantations and challenged with a *C. coli* patient isolate (circles) or vehicle as mock controls (diamonds) on days 0 and 1 by gavage. On day 21 post-infection, TNF-α (**A**,**C**) and IFN-γ (**B**,**D**) concentrations were determined in supernatants of ex vivo biopsies derived from colon (**A**,**B**) and mesenteric lymph nodes (MLN; (**C**,**D**)). Medians (black bars), significance levels (*p*-values calculated by the Kruskal–Wallis test and Dunn’s post-correction), and total numbers of mice under investigation (in parentheses) are shown. Definite outliers were removed after identification by the Grubb’s test (α = 0.001). Results from four experiments were pooled.

Next, we determined pro-inflammatory cytokine secretion in distinct compartments of the intestinal tract. At day 21 post-*C. coli* infection, only mice harboring a human intestinal microbiota displayed higher tumor necrosis factor (TNF)-α concentrations in ex vivo biopsies derived from colon and mesenteric lymph nodes (MLN) draining the inflamed intestines as compared to mock counterparts (*p* < 0.001; Figure 8A,C), which also held true for interferon (IFN)-γ measured in the MLN (*p* < 0.05; Figure 8D). Hence, *C. coli* infection of mice with a human, but not murine, intestinal microbiota induced apoptotic cell and pro-inflammatory immune responses within the intestinal tract.

### 2.5. Extra-Intestinal Including Systemic Pro-Inflammatory Immune Responses upon Peroral C. coli Infection of Microbiota-Depleted IL-10^−/−^ Mice That Had Been Subjected to Human or Murine Fecal Microbiota Transplantations

**Figure 9:**
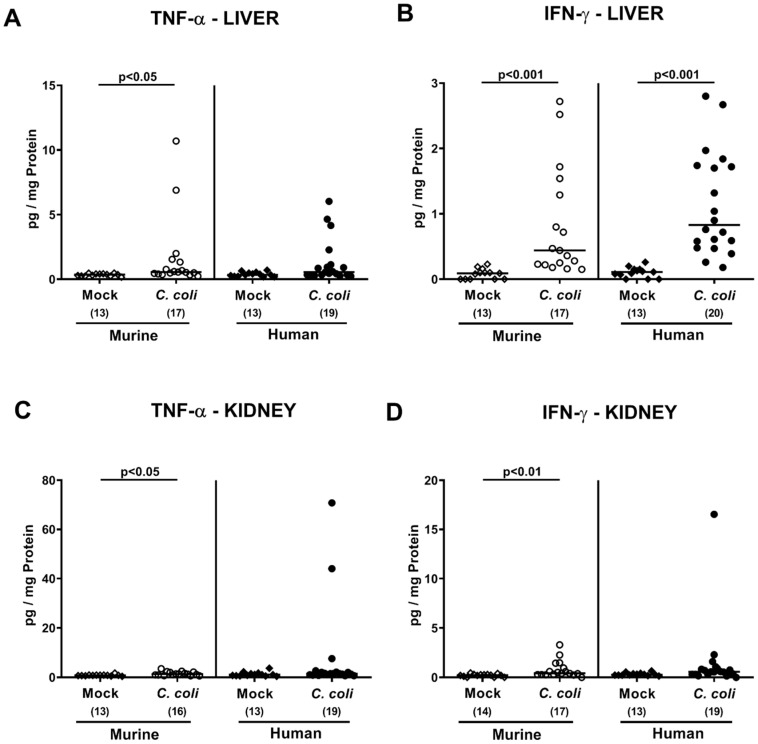
Extra-intestinal secretion of pro-inflammatory cytokines upon oral *C. coli* infection of microbiota-depleted IL-10^−/−^ mice that had been challenged with human or murine fecal microbiota transplantations. On days -7, -6, and -5, mice were subjected to murine (open symbols) or human (closed symbols) fecal microbiota transplantations and challenged with a *C. coli* patient isolate (circles) or vehicle as mock controls (diamonds) on days 0 and 1 by gavage. On day 21 post-infection, TNF-α (**A**,**C**) and IFN-γ (**B**,**D**) concentrations were determined in supernatants of ex vivo biopsies derived from liver (**A**,**B**) and kidney (**C**,**D**). Medians (black bars), significance levels (*p*-values calculated by the Kruskal–Wallis test and Dunn’s post-correction), and total numbers of mice under investigation (in parentheses) are shown. Definite outliers were removed after identification by the Grubb’s test (α = 0.001). Results from four experiments were pooled.

Next, we asked whether the observed *C. coli*-induced inflammatory effects in mice with a human intestinal microbiota were restricted to the intestinal tract or also effective in extra-intestinal, including systemic, compartments. At day 21 p.i., increased TNF-α concentration could be assessed in liver and kidneys of mice with a murine, but not human, intestinal microbiota (*p* < 0.05–0.01 vs. mock; Figure 9A,C,D), whereas pathogen-induced increases in hepatic IFN-γ concentrations were from the microbiota independent (*p* < 0.001 vs. mock; Figure 9B).

**Figure 10:**
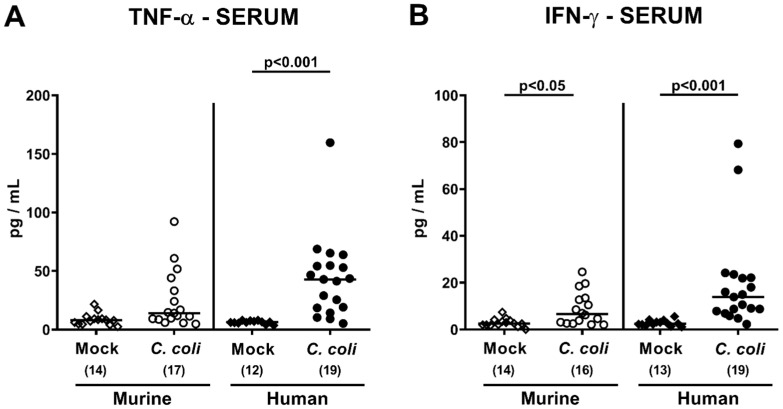
Systemic secretion of pro-inflammatory cytokines upon oral *C. coli* infection of microbiota-depleted IL-10^−/−^ mice that had been challenged with human or murine fecal microbiota transplantations. On days -7, -6, and -5, mice were subjected to murine (open symbols) or human (closed symbols) fecal microbiota transplantations and challenged with a *C. coli* patient isolate (circles) or vehicle as mock controls (diamonds) on days 0 and 1 by gavage. On day 21 post-infection, (**A**) TNF-α and (**B**) IFN-γ concentrations were determined in serum samples. Medians (black bars), significance levels (*p*-values calculated by the Kruskal–Wallis test and Dunn’s post-correction), and total numbers of mice under investigation (in parentheses) are shown. Definite outliers were removed after identification by the Grubb’s test (α = 0.001). Results from four experiments were pooled.

In the systemic compartment, *C. coli*-induced inflammatory sequelae could be assessed in mice with either microbiota composition: Whereas, at day 21 p.i., increased TNF-α concentrations could only be measured in serum samples taken from human microbiota-associated mice (*p* < 0.00 vs. mock; Figure 10A), IFN-γ concentrations were higher in infected versus mock challenged controls of either intestinal microbiota (*p* < 0.05–0.001; Figure 10B).

We further asked whether viable *C. coli* might have translocated from the infected intestines to respective extra-intestinal and even systemic tissue sites. However, pathogenic cells could neither be isolated from liver nor from kidney ex vivo biopsies. In addition, all cardiac blood samples remained culture-negative.

Hence, whereas in the intestinal tract inflammatory sequelae of *C. coli* infection could exclusively be observed in mice with a human intestinal microbiota, extra-intestinal, including systemic, pro-inflammatory responses upon pathogen challenge could be assessed in mice of either microbiota.

## 3. Discussion

Based on our recent findings that *C. coli*—as opposed to *C. jejuni*—are able to overcome the physiological colonization resistance of mice exerted by the murine intestinal microbiota composition, and therefore, can stably establish within the gastrointestinal tract of conventionally colonized wildtype mice [24], here, for the first time, we addressed whether the host-specific human as compared to murine microbiota composition impacts the outcome of campylobacteriosis induced by *C. coli* in microbiota-depleted IL-10^−/−^ mice representing a reliable clinical experimental model for *C. jejuni* infection and induced inflammation [19]. During the entire observation period (i.e., 21 days) after *C. coli* infection of IL-10^−/−^ mice that had been re-associated with a murine or human intestinal microbiota upon peroral FMT, comparable pathogen loads could be determined in the intestinal tract. One could argue that the respective fecal donor microbiota might not have fully established in the murine host. Our cultural analyses and culture-independent molecular approaches additionally assessing fastidious and non-cultivable bacteria revealed, however, that the host-specific differences in numbers of the most common intestinal bacterial groups, genera, and species between respective fecal donor suspensions could also be observed in the intestinal tract of IL-10^−/−^ mice after establishment of the complex human versus murine intestinal microbiota following peroral FMT. Even though the methods applied here have their limitations regarding the possibilities to provide a complete picture of the commensal ecological conditions within the gut lumen, the combinatory approach of quantitative “culturomics” plus 16S rRNA-based analyses have been proven highly reliable for a comprehensive survey of differences in host-specific intestinal microbiota compositions [15,16,24,26,27,28,29,30,31,32,33,34]. One needs to take into account, however, that during processing of the fecal donor samples, including freezing and thawing, individual bacterial strains might have been reduced and not fully established within the intestinal tract upon FMT [27,30,35]. Nevertheless, it is rather the gut luminal milieu provided by the orchestrated interplay between the vast majority of the complex intestinal microbiota than by individual players accounting for the host-specific differences of biological impact. Therefore, it would be utmost appreciable in future studies to assess differences in the intraluminal milieu by metabolomic and/or proteomic approaches, for instance, in order to obtain further insights into the underlying molecular mechanisms of pathogen–host interactions.

Despite comparable intestinal *C. coli* loads in mice harboring a human versus a murine intestinal microbiota, pathogen-induced clinical signs such as fecal blood could more frequently be observed in the former as compared to the latter, especially during the early phase of infection. Moreover, the host microbiota-dependent differences in *C. coli*-induced sequelae were also true on the microscopic level given that only mice harboring a human as opposed to a murine microbiota presented with (i) pronounced colonic epithelial apoptosis, (ii) higher numbers of recruited innate and adaptive immune cells in their large intestinal mucosa and lamina propria, and (iii) an enhanced secretion of pro-inflammatory cytokines such as TNF-α in the colon and MLN draining the infected intestines. The attempt to correlate the observed microbiota-dependent effects during *C. coli* infection with distinct bacterial species appears to be challenging, and, literally, rather a search for the needle in the haystack. However, when comparing both the donor suspensions of human and murine origin and the fecal samples derived from human and murine intestinal microbiota-associated mice, the former contained higher enterobacterial loads as compared to the latter, for instance. Our previous studies revealed that acute as well as chronic inflammatory conditions within the intestinal tract of different etiologies are accompanied by an overgrowth of the inflamed gut lumen with enterobacterial commensals including *Escherichia coli*, which are involved in the initiation and also the exacerbation of the inflammatory immune responses in a Toll-like receptor-4 (TLR-4)-dependent fashion [34,36,37,38,39,40,41,42,43]. It is thus tempting to speculate that interactions (the “crosstalk”) between *C. coli* on the pathogen side and Gram-negative species such as *Escherichia coli* on the commensal gut bacterial side might be responsible for the observed sequelae of *C. coli* infection in IL-10^−/−^ mice harboring a human intestinal microbiota [44].

Interestingly, whereas in the intestinal tract, inflammatory sequelae of *C. coli* infection were exclusively observed in mice harboring a human intestinal microbiota, extra-intestinal, including systemic, pro-inflammatory responses upon pathogen challenge could be assessed in mice of either microbiota. To further unravel these rather unexpected results, we assessed whether viable *C. coli* might have translocated from the gastrointestinal tract to extra-intestinal and systemic tissue sites, but were unable to isolate any bacteria from liver, kidneys, and blood. Nevertheless, soluble cell wall constituents such as *C. coli*-LOS might have translocated to respective compartments, resulting in the observed inflammatory immune responses.

In summary, our present study revealed that peroral *C. coli* infection results in pronounced clinical signs, apoptotic, and pro-inflammatory immune responses in the intestinal tract of IL-10^−/−^ mice harboring a human intestinal microbiota, whereas IL-10^−/−^ mice with a murine intestinal microbiota were stably colonized by the pathogen, but protected from disease manifestations. Given that murine FMT treatment could effectively dampen pathogen-induced apoptotic epithelial and pro-inflammatory immune responses in the large intestines of *C. jejuni*-infected microbiota-depleted, as well as of “humanized”, IL-10^−/−^ mice [27,31], these findings collectively provide strong evidence that microbiota modifications by pre- or probiotics, for instance, might open novel avenues for future treatment strategies in the combat of campylobacteriosis in humans.

## 4. Materials and Methods

### 4.1. Ethics Statement

All mouse experiments were performed in accordance to the European Guidelines for animal welfare (2010/63/EU) after being approved by the commission for animal experiments (“Landesamt für Gesundheit und Soziales”, LaGeSo, Berlin, registration number G0247/16). The clinical conditions of animals were assessed twice a day.

### 4.2. Generation of Microbiota-Depleted Mice

IL-10^−/−^ mice with a C57BL/6J background were bred and maintained under specific pathogen-free conditions in the identical unit of the Forschungseinrichtungen für Experimentelle Medizin (Charité-University Medicine Berlin). Under standard conditions (i.e., 22–24 °C temperature, 55% ± 15% humidity, 12 h light/12 h dark cycle), animals were kept in cages with filter tops within an experimental semi-barrier. Mice had access to autoclaved water and chow (food pellets: ssniff R/M-H, V1534-300, Sniff, Soest, Germany) ad libitum. To overcome physiological colonization resistance, mice were treated with broad-spectrum antibiotic compounds rendering them secondary abiotic, as described earlier [16,36]. Briefly, immediately after weaning, 3-week-old mice were treated with an antibiotic cocktail consisting of ampicillin plus sulbactam (1 g/L; Dr. Friedrich Eberth Arzneimittel, Ursensollen, Germany), vancomycin (500 mg/L; Hikma Pharmaceuticals, London, UK), ciprofloxacin (200 mg/L; Fresenius Kabi, Bad Homburg, Germany), imipenem (250 mg/L; Fresenius Kabi), and metronidazole (1 g/L; B. Braun, Melsungen, Germany) dissolved in autoclaved drinking water (ad libitum) [36]. To ensure antimicrobial washout, the antibiotic cocktail was withdrawn and replaced by autoclaved water three days prior to the initial FMT.

### 4.3. Fecal Microbiota Transplantations

On days -7, -6, and -5, sex- and age-matched microbiota-depleted IL-10^−/−^ mice (three-month-old littermates) were perorally subjected to peroral murine or human FMT by gavage, as described earlier in more detail [16,29,30,35,45]. Briefly, fecal samples (lacking enteropathogenic parasites, viruses, and bacteria) were derived from five healthy human donors or from 3-month-old C57BL/6 mice (3 female, 2 male murine donors), suspended in phosphate buffered saline (PBS; Thermo Fisher Scientific, Waltham, MA, USA), and aliquots stored at −80 °C. For FMT, respective fecal aliquots were thawed, pooled, and applied to mice by gavage (total volume of 0.3 mL). For stable establishment of the human and murine intestinal microbiota in the gastrointestinal tract of the murine host, the *C. coli* challenge started one week post-FMT.

### 4.4. C. coli Infection, Colonisation Properties, and Translocation

The used *C. coli* strain had been isolated from a patient with bloody diarrhea (kindly provided by Dr. Torsten Semmler, Robert-Koch-Institute Berlin, Berlin, Germany). Mice were perorally challenged with 10^8^ *C. coli* cells by gavage (in 0.3 mL PBS) on days 0 and 1, as stated earlier [24,44].

To assess gastrointestinal colonization properties, *C. coli* were enumerated in fecal samples at distinct time points post-infection by culture and additionally in gastrointestinal luminal samples including colon, ileum, duodenum, and stomach on the day of sacrifice, as reported previously [24,44]. Briefly, serial dilutions of fecal samples were plated on Karmali agar plates and on Columbia agar plates containing 5% sheep blood (Oxoid, Wesel, Germany) and incubated under microaerophilic conditions in a jar for 48 h at 37 °C. For assessing bacterial translocation, ex vivo biopsies taken from the kidneys, liver, and MLN were homogenized in sterile PBS and cultivated on respective agar plates, whereas *C. coli* were isolated from cardiac blood [24,44]. The *C. coli* detection limit was ≈100 CFU/g.

### 4.5. Analyses of the Intestinal Microbiota Composition

In order to quantitatively assess the microbiota composition in fecal human and murine donor suspensions and colonic luminal contents, samples were homogenized in sterile PBS and enumerated from serial dilutions on respective solid media and grown at 37 °C for at least two days under aerobic, microaerobic, and anaerobic conditions, as described earlier [37]. For culture-independent (molecular) intestinal microbiota analysis, the total genomic DNA was extracted from the donor suspensions and colonic luminal samples as reported earlier [37]. The main bacterial groups within the human and murine intestinal microbiota were surveyed by quantitative real-time polymerase chain reaction (qRT-PCR) with species-, genera-, or group-specific 16S rRNA gene primers (Tib MolBiol, Berlin, Germany), as described previously [45,46].

### 4.6. Clinical Conditions

Before initial FMT and *C. coli* challenge as well as after bacterial infections, the clinical status of mice was surveyed applying a standardized, cumulative clinical scoring system (maximum 12 points), which took into account the overall clinical aspects (0: normal; 1: ruffled fur; 2: less locomotion; 3: isolation; 4: severely compromised locomotion, wasting, pre-final aspect), the occurrence of blood in feces (0: no blood; 2: microscopic detection of blood by the Guajac method using Haemoccult, Beckman Coulter/PCD, Germany; 4: macroscopic blood visible), and diarrhea (0: formed feces; 2: pasty feces; 4: liquid feces), as described previously [47]. The fecal blood positivity rate was calculated by dividing the number of macroscopically and/or microscopically fecal blood-positive mice by the total number of analyzed mice.

### 4.7. Sampling Process

On day 21 p.i., mice were sacrificed by CO_2_ asphyxiation. Ex vivo biopsies were taken under sterile conditions from liver, kidneys, MLN, and colon, in addition to luminal gastrointestinal samples from the stomach, duodenum, ileum, and colon. Blood was collected by heart puncture. Samples were taken from each mouse for microbiological, immunohistopathological, and immunological analyses in parallel.

### 4.8. Histopathology and Immunohistochemistry

Histopathological analyses were performed in colonic ex vivo biopsies that had been immediately fixed in 5% formalin and embedded in paraffin. Sections (5 μm) were stained with hematoxylin and eosin (H&E), examined by light microscopy (100× magnification), and histopathological changes in the large intestines were quantitatively assessed applying an established histopathological scoring system as reported previously [25]: Score 1: minimal inflammatory cell infiltrates in the mucosa with intact epithelium, Score 2: mild inflammatory cell infiltrates in the mucosa and submucosa with mild hyperplasia and mild goblet cell loss, Score 3: moderate inflammatory cell infiltrates in the mucosa with moderate goblet cell loss, and Score 4: marked inflammatory cell infiltration into in the mucosa and submucosa with marked goblet cell loss, multiple crypt abscesses, and crypt loss.

### 4.9. Immunohistochemistry

In situ immunohistochemical analyses were carried out in colonic ex vivo biopsies immediately fixed in 5% formalin and embedded in paraffin, as previously described [41,48,49,50]. For detection of apoptotic and proliferating epitshelial cells, macrophages/monocytes, T lymphocytes, regulatory T cells, and B lymphocytes, 5 μm thin colonic paraffin sections were stained with primary antibodies directed against cleaved caspase 3 (Asp175, Cell Signaling, Beverly, MA, USA, 1:200), Ki67 (TEC3, Dako, Glostrup, Denmark, 1:100), F4/80 (# 14-4801, clone BM8, eBioscience, San Diego, CA, USA, 1:50), CD3 (#N1580, Dako, 1:10), FOXP3 (clone FJK-165, #14-5773, eBioscience, 1:100), and B220 (No. 14-0452-81, eBioscience; 1:200), respectively. Positively stained cells were enumerated using light microscopy, and the average number within at least 6 high-power fields (HPF, 0.287 mm^2^, 400× magnification) was recorded by an unbiased investigator.

### 4.10. Pro-Inflammatory Mediator Measurements in Intestinal, Extra-Intestinal, and Systemic Compartments

Colonic explants were cut longitudinally, rinsed in PBS, and strips of ≈1 cm^2^ tissue and ex vivo biopsies taken from liver (approximately 1 cm^3^), kidney (one half), and MLN (3 lymph nodes) were placed into 24 flat-bottom-well culture plates (Thermo Fisher Scientific, Waltham, MA, USA) supplemented with 500 μL serum-free RPMI 1640 medium (Thermo Fisher Scientific, Waltham, MA, USA) as well as penicillin (100 U/mL) plus streptomycin (100 µg/mL; Biochrom, Berlin, Germany). After incubation for 18 h at 37 °C, respective culture supernatants as well as serum samples were tested for TNF-α and IFN-γ by the Mouse Inflammation Cytometric Bead Array (CBA; BD Biosciences, Heidelberg, Germany) on a BD FACSCanto II flow cytometer (BD Biosciences, Heidelberg, Germany).

### 4.11. Statistical Analysis

Medians and levels of significance were calculated utilizing the Mann–Whitney test (GraphPad Prism v8, San Diego, CA, USA) for pairwise comparisons of not normally distributed data, and using the Kruskal–Wallis test with Dunn’s post-correction for multiple comparisons, as indicated. Two-sided probability (*p*) values ≤ 0.05 were considered significant. Definite outliers were removed after being identified by the Grubb’s test (α = 0.001). Data were pooled from four independent experiments.

## Supplementary Materials

The following are available online at https://www.mdpi.com/2076-0817/9/10/804/s1, Figure S1: Experimental set-up. Figure S2: Kinetic survey of overall clinical conditions following peroral *C. coli* challenge of microbiota-depleted IL-10^−/−^ mice that had been subjected to murine or human fecal microbiota transplantations. Figure S3: Representative photomicrographs depicting (A) histopathological changes in hematoxylin and eosin (H&E)-stained colonic paraffin sections, and average numbers of (B) apoptotic (caspase3+, Casp3+) and (C) proliferating (Ki67+) epithelial cells, of (D) macrophages and monocytes (F4/80+), (E) T lymphocytes (CD3+), (F) regulatory T cells (FOXP3+), and (G) B lymphocytes (B220+) in the colonic mucosa and lamina propria (from at least 6 high-power fields), as quantitatively determined in immunohistochemically stained colonic paraffin sections (100× magnification, scale bar 100 μm).
