# Supplementary material for: The Host-Specific Intestinal Microbiota Composition Impacts Campylobacter coli Infection in a Clinical Mouse Model of Campylobacteriosis
Source: Pathogens. 2020 Sep 29;9(10):804. doi: 10.3390/pathogens9100804 (PMC7600086; doi:10.3390/pathogens9100804)

A

Histopathology (H&E) - COLON

Mock
Murine

C. coli
Murine

Mock
Human

C. coli
Human

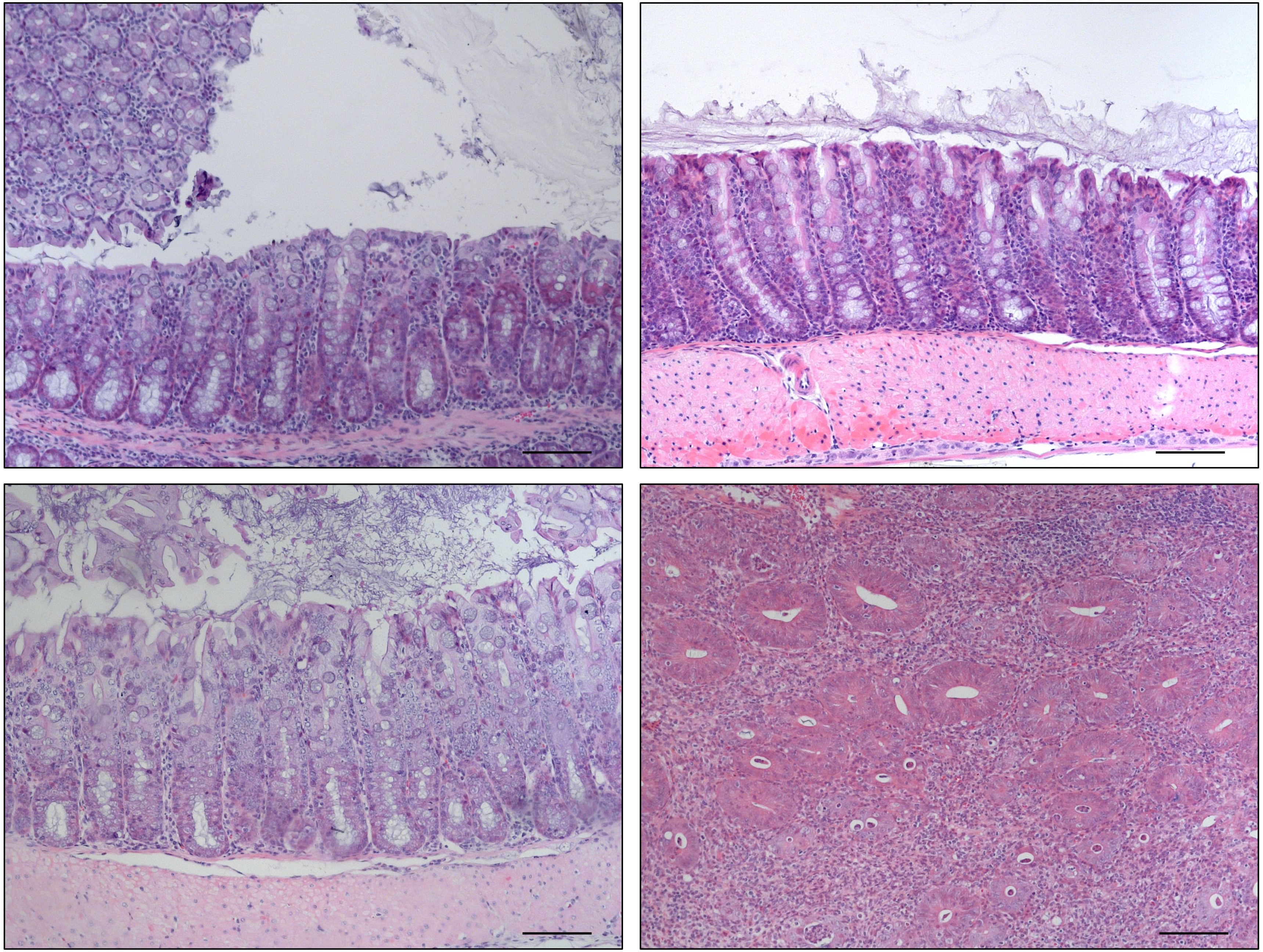

B

Apoptotic Cells (Casp3+) - COLON

Mock Murine

Mock Human

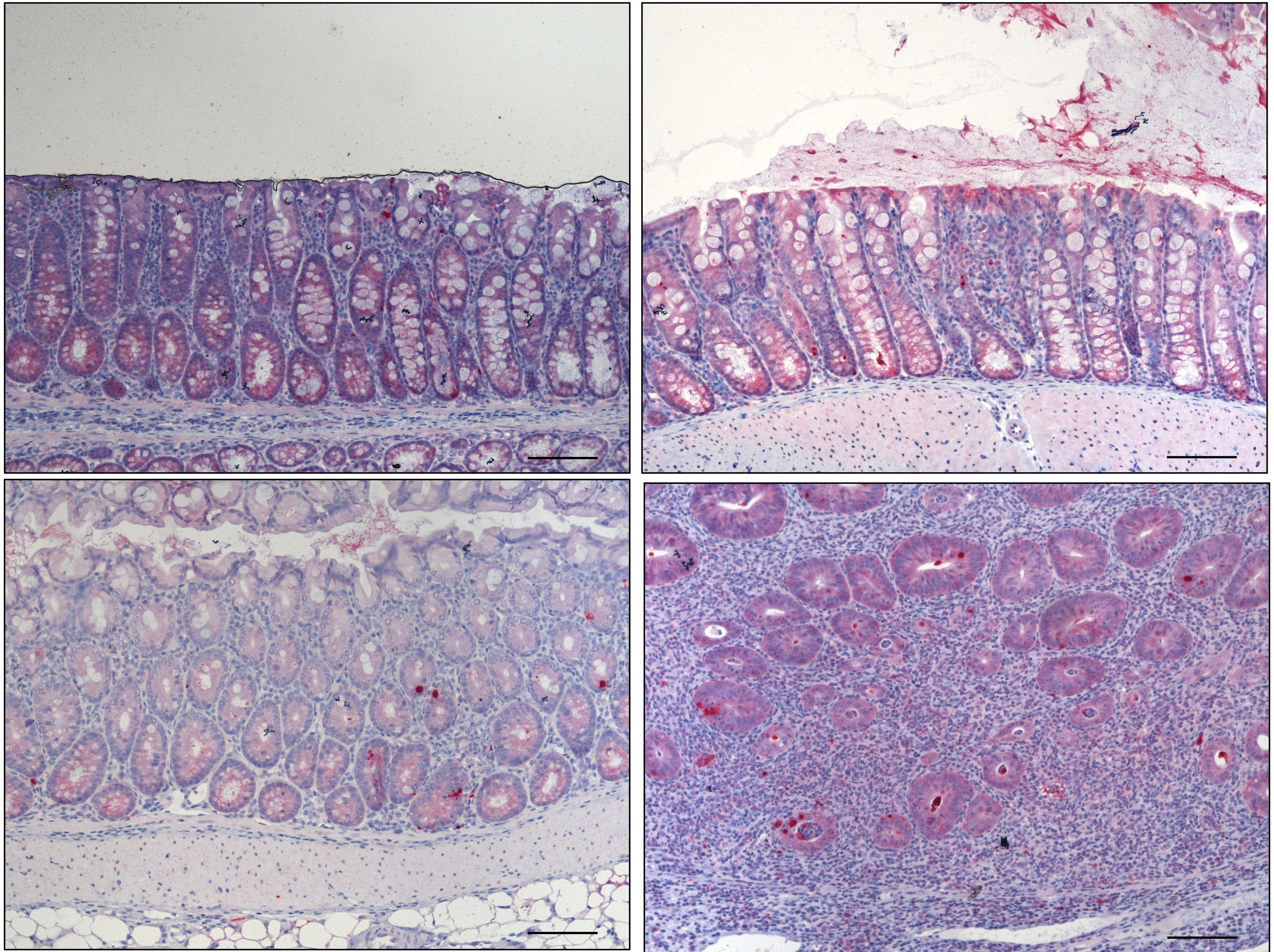

***C. coli* Murine**

***C. coli* Human**

C

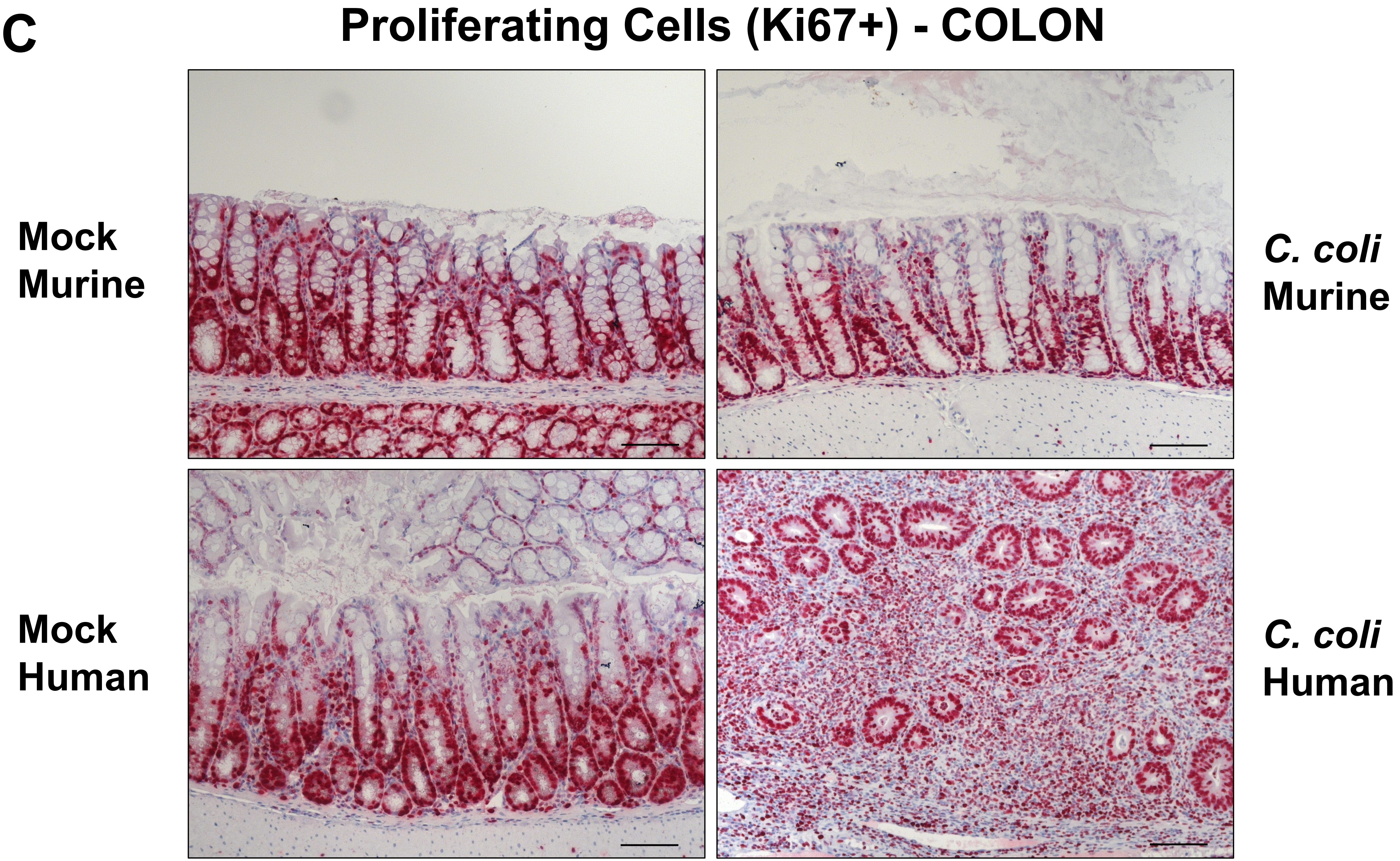
Proliferating Cells (Ki67+) - COLON
Mock
Murine
C. coli
Murine
Mock
Human
C. coli
Human

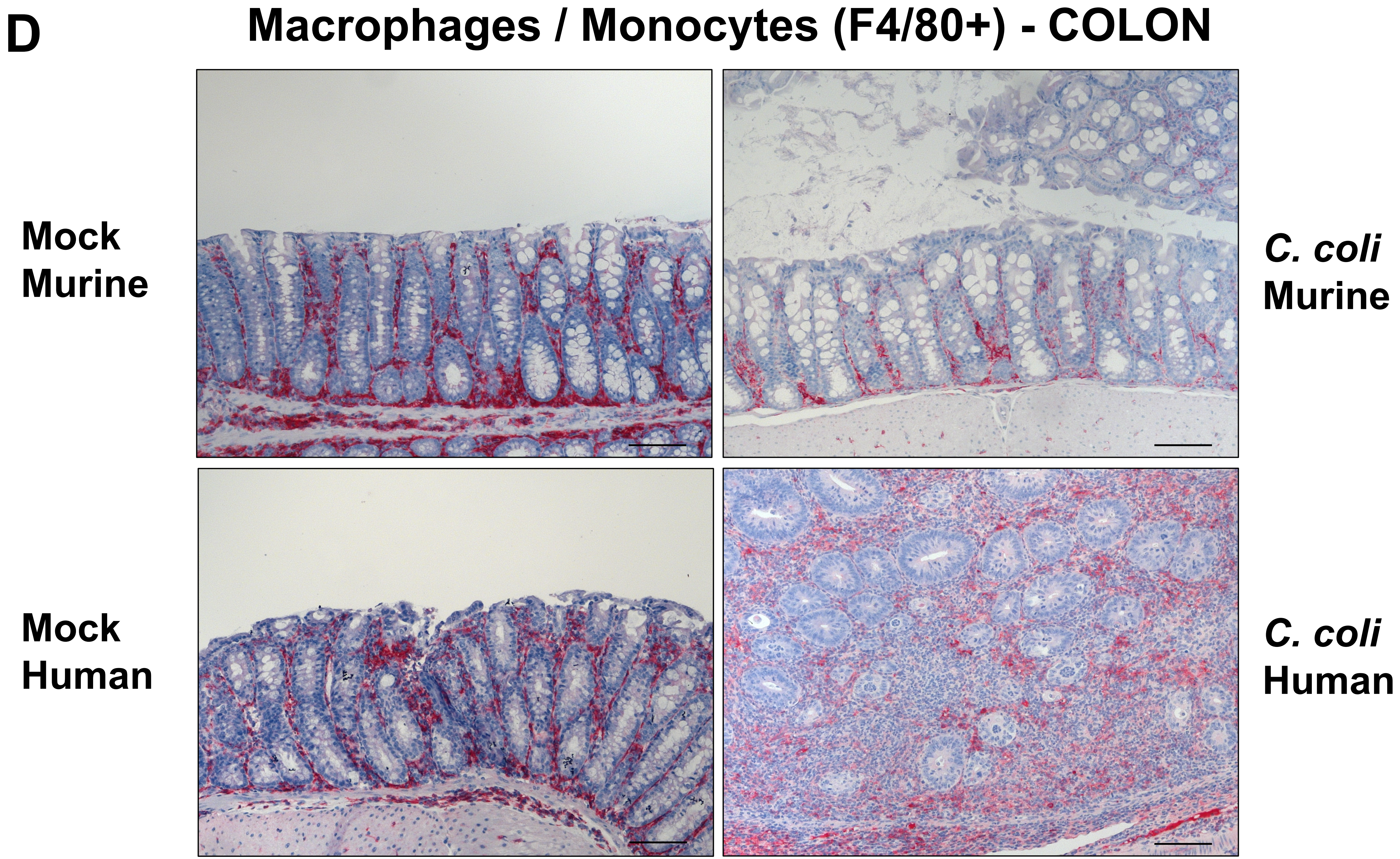
D
Macrophages / Monocytes (F4/80+) - COLON
Mock
Murine
C. coli
Murine
Mock
Human
C. coli
Human

E

T Lymphocytes (CD3+) - COLON

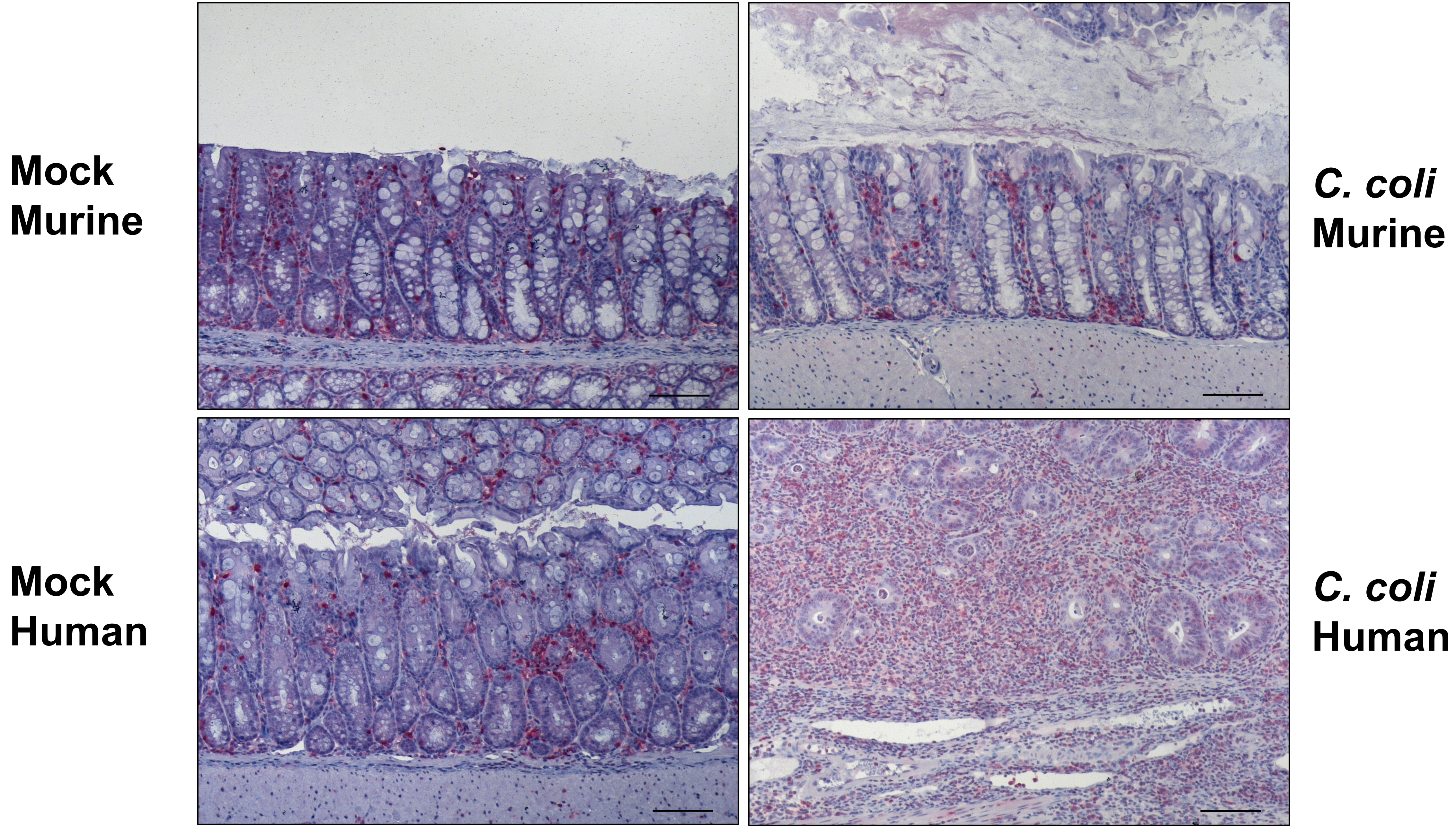

F

Regulatory T Cells (FOXP3+) - COLON

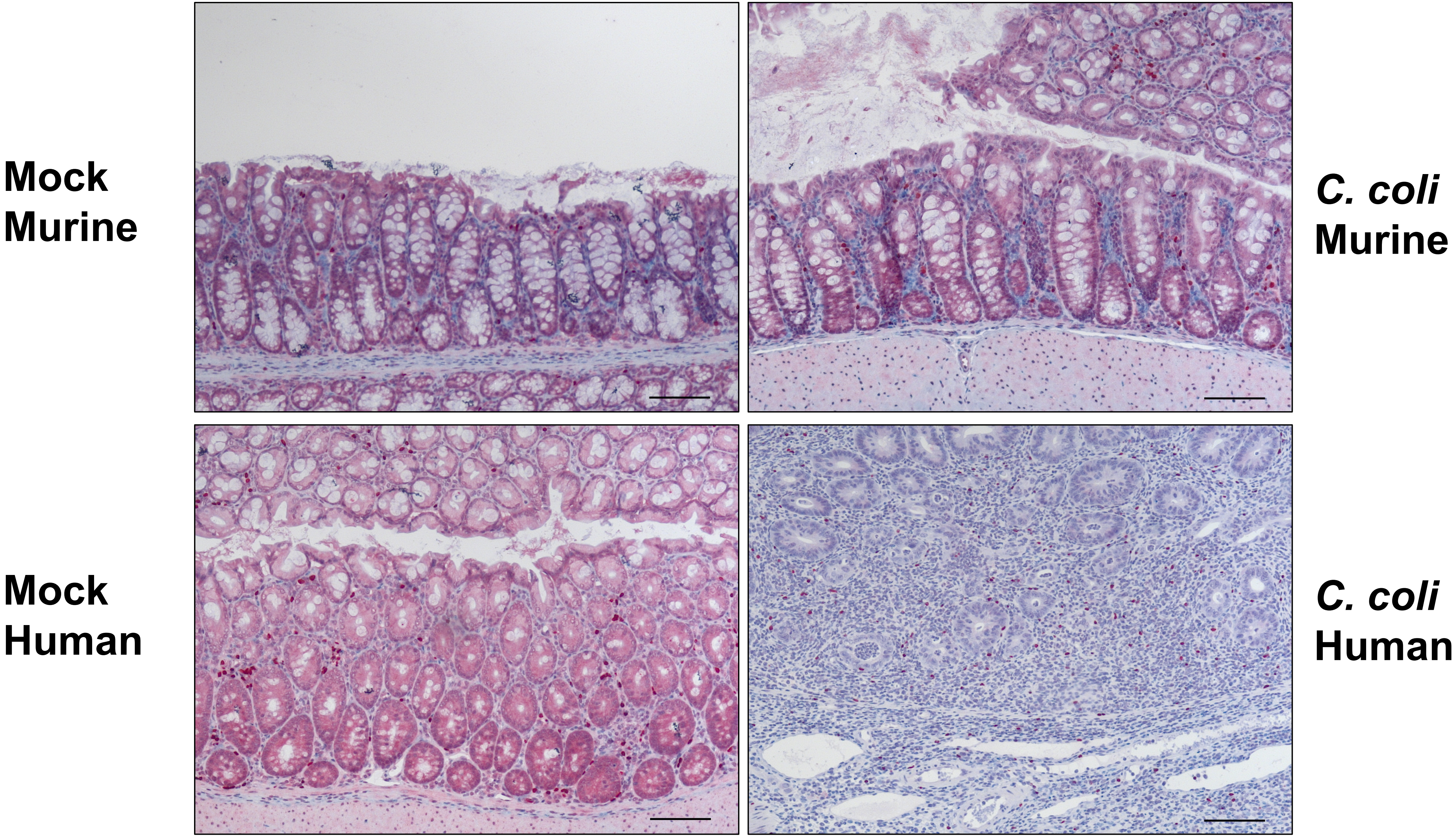

G

B Lymphocytes (B220+) - COLON

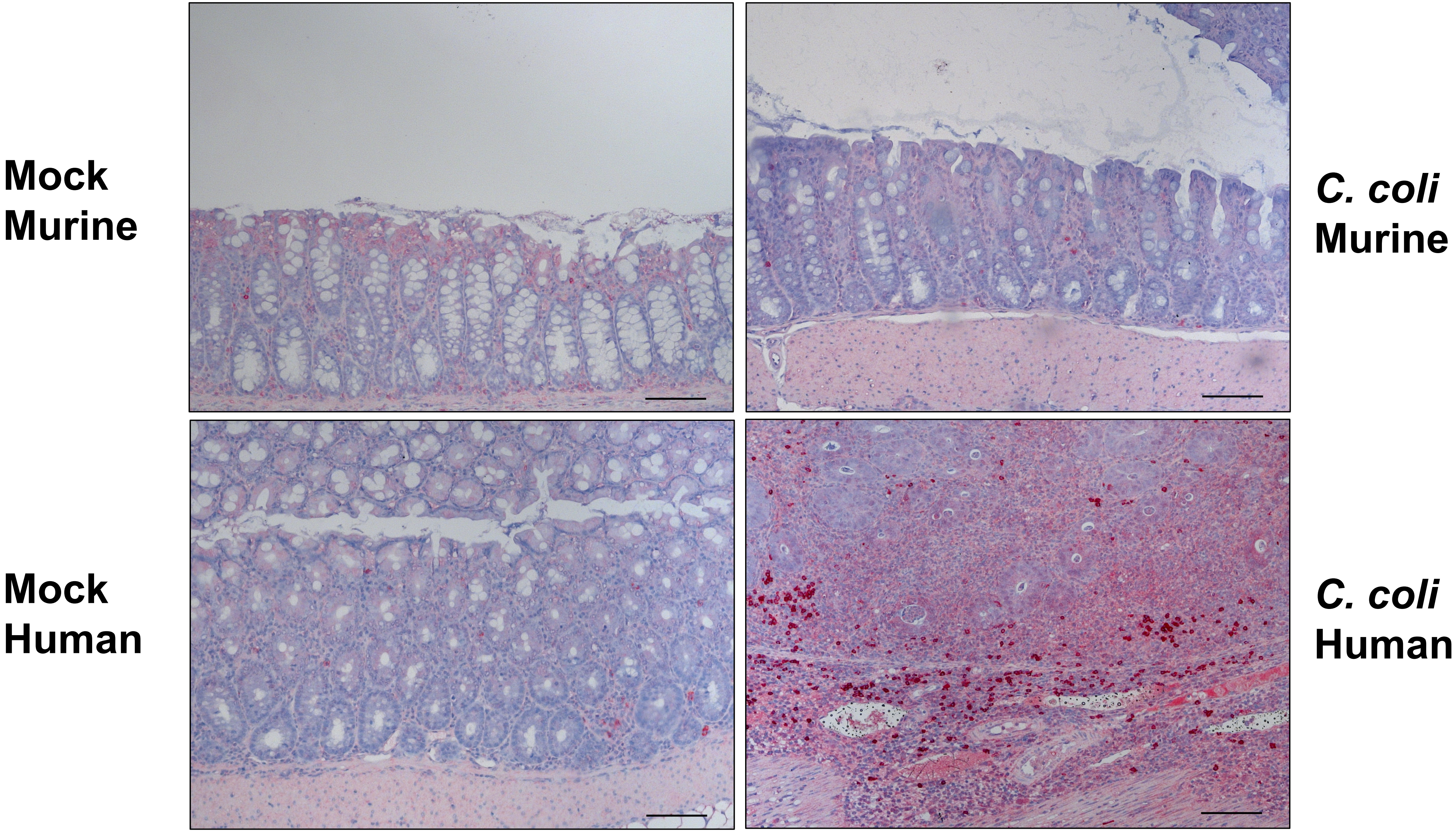

Supplement: Supplementary file 1 [file pathogens-09-00804-s001.zip › FigureS3_COLON_PICS_210820.pdf]
