# Supplementary figures and images for: The Host-Specific Intestinal Microbiota Composition Impacts Campylobacter coli Infection in a Clinical Mouse Model of Campylobacteriosis
Source: Pathogens. 2020 Sep 29;9(10):804. doi: 10.3390/pathogens9100804 (PMC7600086; doi:10.3390/pathogens9100804)

# Experimental Setup

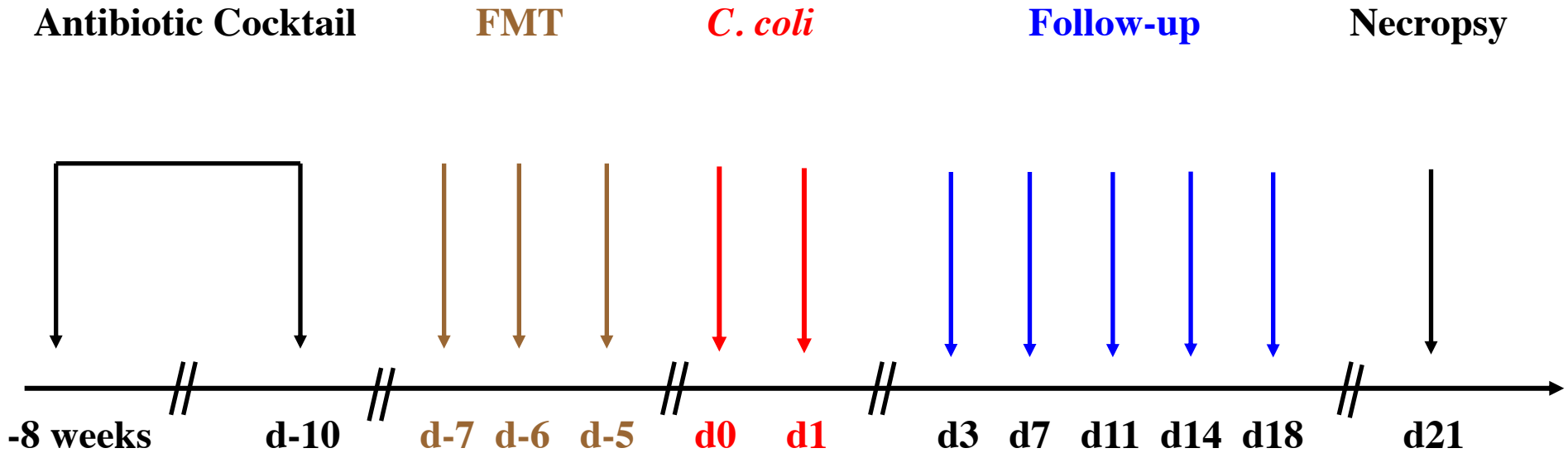

Supplement: Supplementary file 1 [file pathogens-09-00804-s001.zip › FigureS1_Setup_310820.pdf]

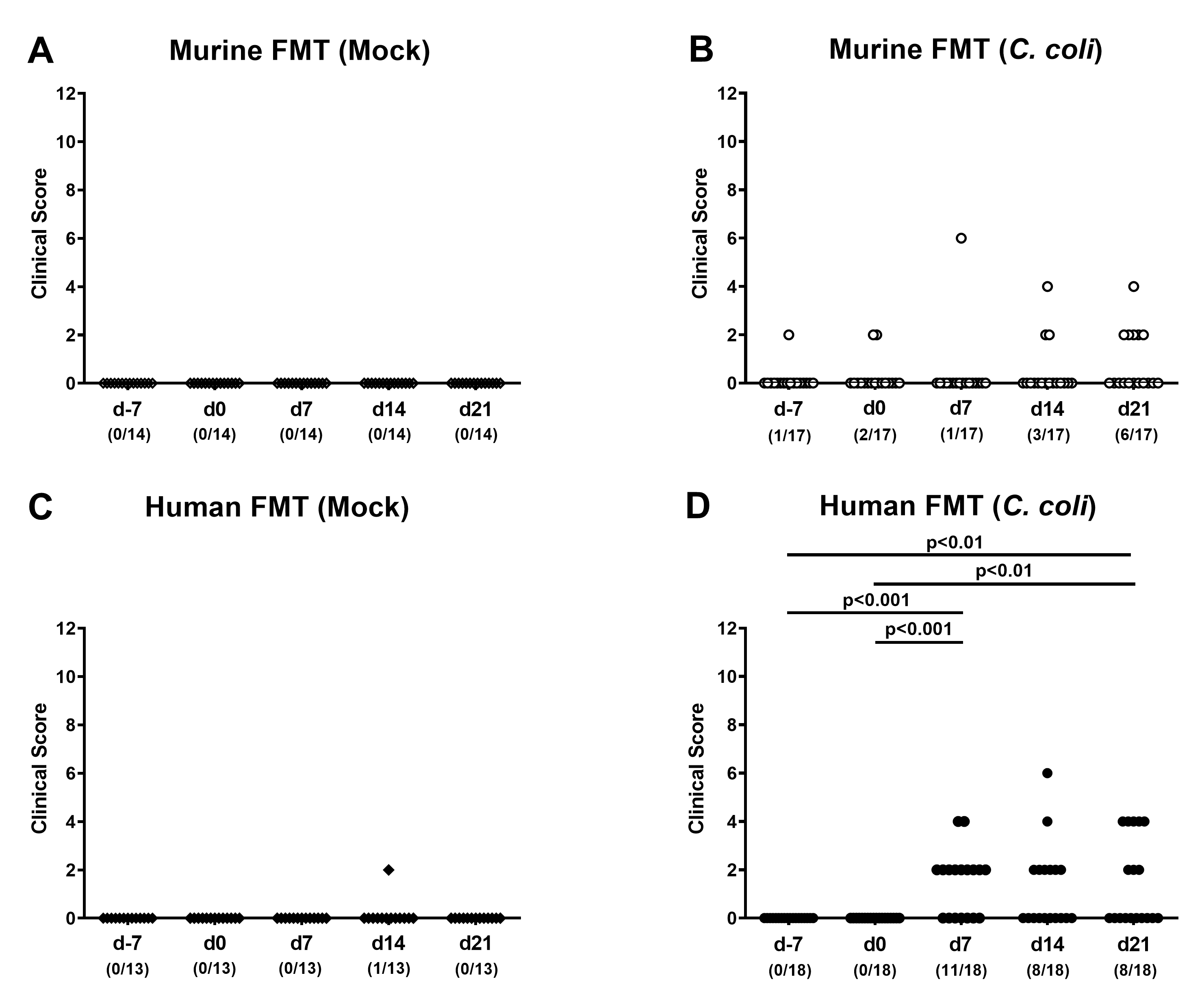

Supplement: Supplementary file 1 [file pathogens-09-00804-s001.zip › FigureS2_REVISED.tiff]
